# The global status and hotspots of research in the field of trans-oral endoscopic thyroidectomy (TOET) from 2008 to 2022

**DOI:** 10.3389/fsurg.2023.1120442

**Published:** 2023-04-27

**Authors:** Ping Li, Hao Qin, Rui Jin, Wuping Zheng, Pingming Fan, Peng-fei Lyu

**Affiliations:** ^1^ Department of Maxillofacial and Ear, Nose and Throat Oncology, Tianjin Medical University Cancer Institute and Hospital, Tianjin, China; ^2^ National Clinical Research Center for Cancer, Tianjin's Clinical Research Center for Cancer, Tianjin, China; ^3^ Key Laboratory of Cancer Prevention and Therapy, Tianjin, China; ^4^ Department of Breast Surgery, The First Affiliated Hospital of Hainan Medical University, Haikou, China; ^5^ Department of Thyroid and Breast Surgery, The Second Affiliated Hospital of Hainan Medical University, Haikou, China

**Keywords:** thyroidectomy, transoral, vestibular approach, research trends, TOET, bibliometrics

## Abstract

**Purpose:**

In this study, the aim was to comprehensively analyze the current status, hotspots, and trends of trans-oral endoscopic thyroidectomy (TOET) through bibliometric analysis and by presenting the field atlas.

**Methods:**

Web of Science Core Collection database was adopted to screen studies regarding TOET published between January 1, 2008 and August 1, 2022. The evaluation covered the criteria total number of studies, keywords, and contributions from countries/regions, institutions, journals, and authors.

**Results:**

A total of 229 studies were covered. *SURGICAL ENDOSCOPY AND OTHER INTERVENTIONAL TECHNIQUES* is the largest publication in the field of TOET. The three countries that contributed the most studies were Korea, China, and the USA. The most frequently occurring core keywords in the field of TOET are vestibular approach, outcomes, experience, safety, robotic thyroidectomy, scar, video-assisted thyroidectomy and quality-of-life. The seven clusters were generated in this study: intraoperative monitoring of the laryngeal return nerve (# 0), learning curve (# 1), postoperative quality of life (# 2), central lymph node dissection and safety (# 3), complications (# 4), minimally invasive surgery (# 5), and robotic surgery (# 6).

**Conclusion:**

The main research topics in the field of TOET place focuses on learning curves, laryngeal nerve monitoring, carbon dioxide gas bolus, chin nerve injury, surgical complications, and surgical safety. In the future, more academics will focus on the safety of the procedure and reducing complications..

## 1. Introduction

There are 586,000 cases of thyroid cancer around the world, and the incidence of thyroid cancer ranked 9th in 2020 (1). Papillary thyroid carcinoma (PTC) has been confirmed as a highly common endocrine malignancy in the world. Existing studies have suggested that the global incidence of thyroid cancer has been rising over the past two decades (2). Surgery has been most frequently used to treat thyroid cancer. However, it is inevitable that conventional open thyroidectomy (COT) will leave visible scars on the neck, such that COT is not preferred widely, in particular young females. Patients with a high expectation of cosmetic results, in particular Asian patients having pronounced scars, cannot accept any small surgical scar left on or near their neck (3). In the last century, minimally invasive and remote access surgical technologies emerged and have been applied to numerous surgical specialties (e.g., thoracic, abdominal, pelvic surgery, as well as head and neck surgery in recent years). A wide variety of scar-free surgical approaches had been developed (e.g., total endoscopic thyroidectomy *via* trans-axillary approach and the areola approach (ETA), as well as the transoral endoscopic thyroidectomy vestibular approach (TOETVA) (4–6). TOETVA, a type of natural orifice translumenal endoscopic surgery (NOTES), has been the most used process in transoral thyroidectomy. Its superiorities involve good function short-term voice outcomes, advantages of central lymph nodes dissection, scar free approaches and low surgical morbidity (7).

Bibliometric research is often adopted to evaluate published research, analyze the characteristics of disciplinary developments, and predict future trends in scientific research. The above research has combined mathematical and statistical methods to identify research areas and then visualized and analyzed the number of publications, authors, institutional countries/regions, journals, themes, and so forth. Network research between some of the research indicators forms a relational network diagram and improve the knowledge structure established on subject clusters. The researcher or practitioner can employ the study to gain valuable insights into the lineage, current characteristics, and future trends in the development of the subject area.

TOET refers to an emerging surgical procedure that is rapidly used by surgeons with good results. In this study, bibliometrics is adopted to present comprehensive assessment of global scientific research in TOET, analyzing TOET's disciplinary characteristics, current hotspots, and future trends. On that basis, enthusiasts and practitioners can gain a quick and in-depth understanding of TOET and more effectively identify hotspots of relevant studies.

## 2. Materials and methods

### 2.1. Materials

The WoSCC database was searched for studies published in a range from January 1, 2008 to August 1, 2022. The keywords employed in the database to be used to search the database comprised (transoral OR vestibular OR oral OR mouth OR transorally OR trans-oral OR “vestibule of the mouth” OR TOET OR TOT OR TOETVA) AND (thyroidectomy OR thyroidectomies OR “thyroid lobectomy”) AND (endoscopic OR endoscopy OR video endoscopic). Two authors evaluated the retrieved studies to exclude irrelevant literature. Areas of disagreement were assessed by the corresponding author. All covered studies were downloaded as plain text files and software analysis was performed for reference, keyword, fundings, abstracts, author, titles, and other research indicators.

A total of 285 English-language document records were achieved for transoral thyroidectomy. Duplicates were removed, and document types (original studies and reviews) were selected. Lastly, 229 articles were retained for the bibliometric analysis through manual review and confirmation by two authors.

### 2.2. Methods

Bibliometric tools [e.g., CiteSpace, Vosviewer, Rstudio (bibliometric package), and gCLUTO software] were adopted to analyze the covered studies for institution, country, journal, author, keywords, as well as references. A bibliographic coupling network was established, and a visual network analysis was conducted on the content of the study.

## 3. Results

### 3.1. General information

**Figure 1:**
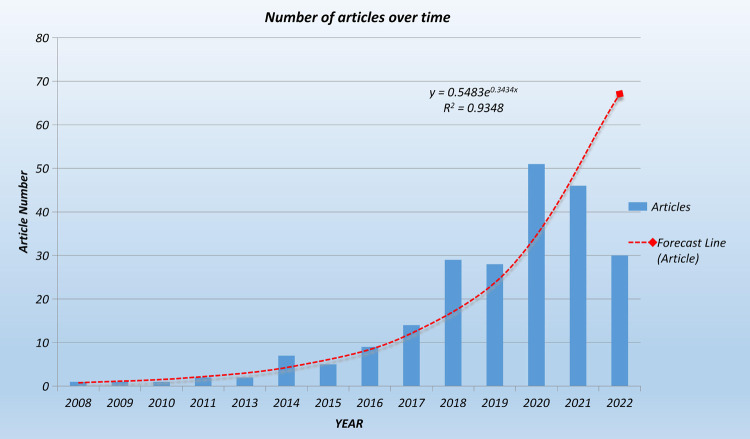
Changes in the number of articles linked to TOET over time. The red dotted line is the forecast line, and the red square represents the forecast document volume in 2022. From 2008 to 2016, the number of studies per year was below 10. Rapid growth from 2017 onward, the number of studies published per year is above 28, reaching 51 in 2020. Based on the prediction curve (red dashed line), it is predicted that 68 studies may come out in 2022.

The time span for TOETVA technology to be applied to people is from 2009 to 2022. 72 journals published studies in the field; and each study was cited an average of 17.28 times per year. In terms of study type, there were 202 original researches and 27 reviews. The number of studies per year is shown in Figure 1.

### 3.2. Analysis of countries, authors, and institutions

**Figure 2:**
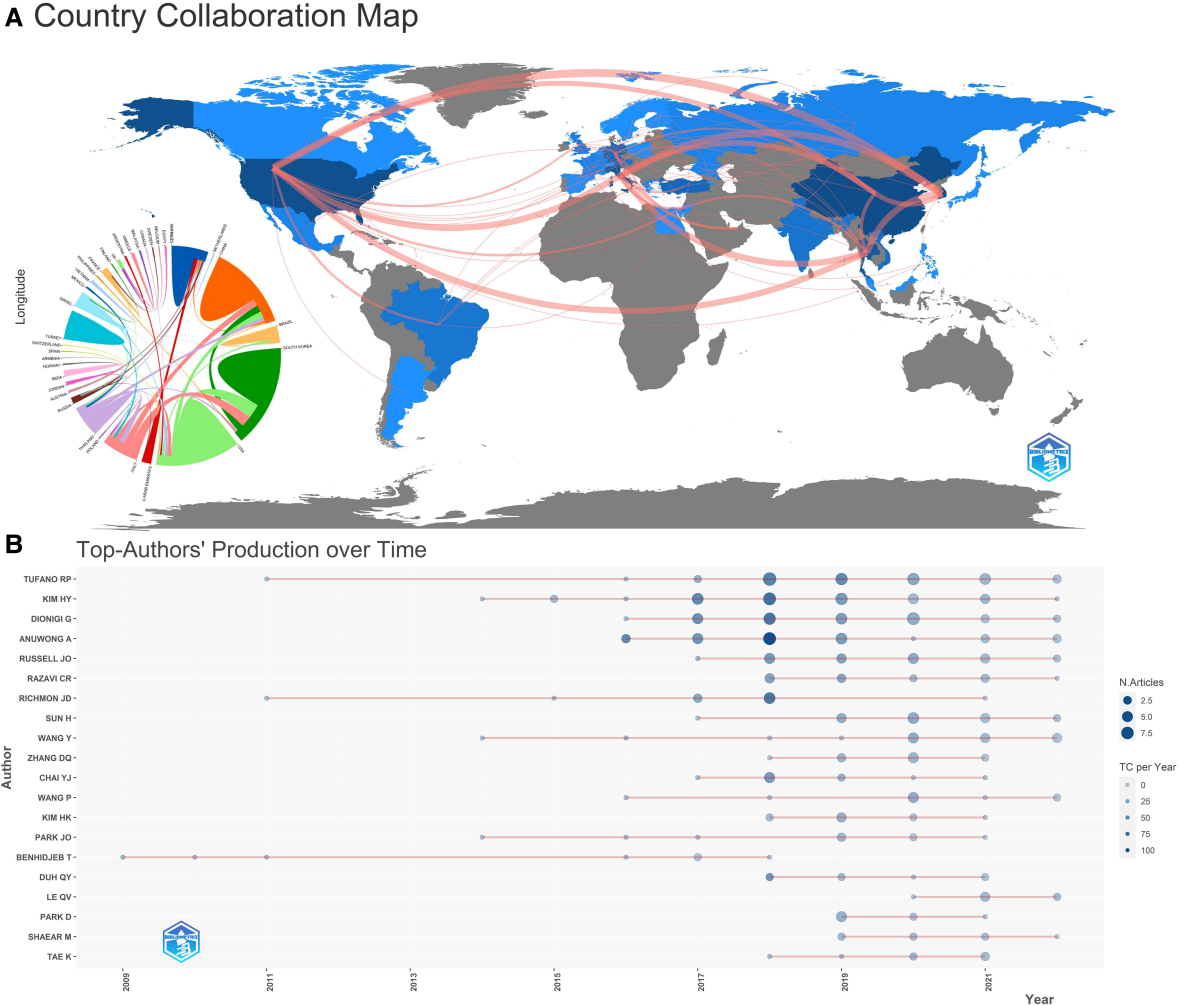
(**A**) national cooperation map. The connection represents the cooperation between countries. The darker the color, the greater the number of articles. (**B**) Top-author's production over time. TC, total citation.

Korea, China, and the United States have outstanding contributions to the development of trans-oral thyroid surgery with 68, 66, and 61 published studies, respectively. Together, the above three countries account for 85.15% of all study publications. Figure 2A shows a map of TOET's national collaboration network with frequent international collaborations.

There are 766 authors from 35 countries contributing to TOETVA. The top 3 most productive authors are Tufano RP, Kim HY and Dionigi G. Tufano RP from the USA published 36 studies, accounting for 15.7% of the total. Kim HY from Thailand published 35 studies (15.2%). Dionigi G from Italy published 32 studies (13.98%). Anuwong A has published 29 studies (12.66%) and is ranked fourth. The top 20 authors' production over time is shown in Figure 2B. The above authors have a high number of studies published and cited after 2017.

**Figure 3:**
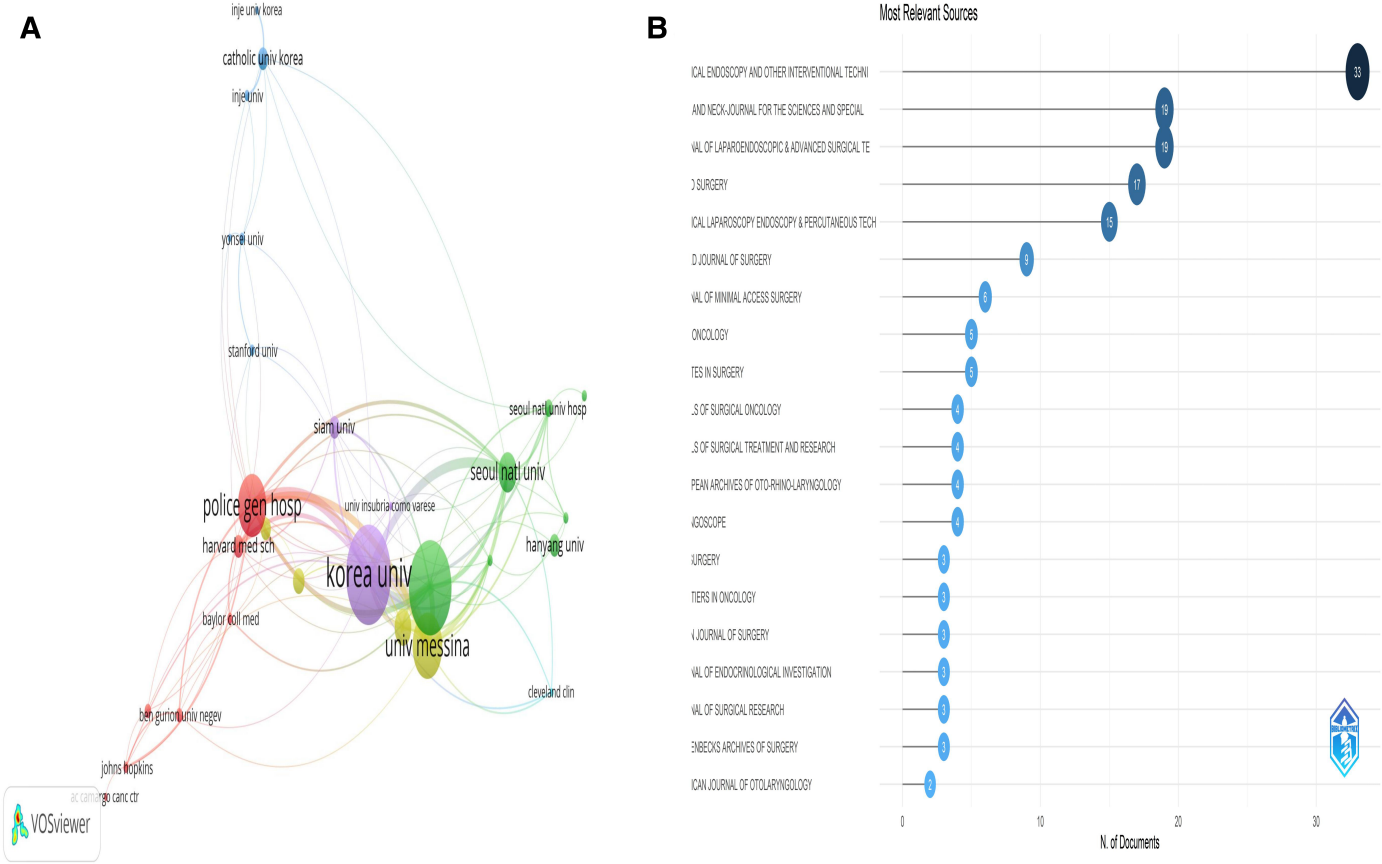
(**A**) organization co-occurrence network diagram. The size of the circle represents the number of documents issued by the organization, and the connection represents the connection between organizations. (**B**) Publications of top 20 related magazines.

In term of TOETVA-related institutions, KOREA UNIV, UNIV MESSINA, Johns Hopkins Univ and POLICE GEN HOSP contribute significantly to TOETVA. Figure 3A is a map of co-occurrence between institutions, with some inter-institutional collaboration. 41 studies were from Johns Hopkins Univ, of which 13 were first author; the most cited institution was Korea Univ with 615 citations. The top 10 contributing institutions to the study about TOETVA is in Table 1.

**Table 1:** Top 10 contributing institutions to the article about TOETVA.

Institution	Article No.	Citations No.	Number of first authors	Number of first author citations
Korea Univ	37	615	11	163
Police Gen Hosp	22	535	6	281
Johns Hopkins Univ	41	472	13	221
Seoul Natl Univ	20	310	6	79
Siam Univ	8	309	1	152
Univ Messina	23	265	1	30
Jinan Univ	5	218	3	112
Harvard Med Sch	8	182	3	22
Univ Calif San Francisco	8	145	2	9

### 3.3. Analysis of journals and top 10 cited studies

*SURGICAL ENDOSCOPY AND OTHER INTERVENTIONAL TECHNIQUES* was the most published journal (*n* = 33), followed by *HEAD AND NECK—JOURNAL FOR THE SCIENCES AND SPECIALTIES OF THE NECK* and *JOURNAL OF LAPAROENDOSCOPIC & ADVANCED SURGICAL TECHNIQUES* (*n* = 19). Figure 3B lists the 20 journals with the highest number of publications.

The most cited study is by Anuwong A from Thailand with 264 citations. Table 2 lists the 10 most cited studies in the field of TOETVA. Among the above 10 studies, 3 studies from Anuwong A with a total of 596 citations are the most cited authors. In addition, four of the 10 most cited studies are from the journal *SURGICAL ENDOSCOPY AND OTHER INTERVENTIONAL TECHNIQUES*, with a total of 463 citations.

**Table 2:** The top 10 most cited articles in TOETVA field.

Rank	Reference	Citation	Journal	IF	Author	Year
1	Transoral Endoscopic Thyroidectomy Vestibular Approach: A Series of the First 60 Human Cases	264	WORLD JOURNAL OF SURGERY	3.504	Anuwong, A	2016
2	Safety and Outcomes of the Transoral Endoscopic Thyroidectomy Vestibular Approa	204	JAMA SURGERY	17.488	Anuwong, A	2018
3	Natural orifice surgery on thyroid gland: totally transoral video-assisted thyroidectomy (TOVAT): report of first experimental results of a new surgical method	140	SURGICAL ENDOSCOPY AND OTHER INTERVENTIONAL TECHNIQUES	3.796	Benhidjeb, T	2009
4	Transoral endoscopic thyroidectomy vestibular approach (TOETVA): indications, techniques and results	128	SURGICAL ENDOSCOPY AND OTHER INTERVENTIONAL TECHNIQUES	3.796	Anuwong, A	2018
5	Trans-Oral Video-Assisted Neck Surgery (TOVANS). A new transoral technique of endoscopic thyroidectomy with gasless premandible approach	115	SURGICAL ENDOSCOPY AND OTHER INTERVENTIONAL TECHNIQUES	3.796	Nakajo, A	2013
6	Thyroidectomy: A novel endoscopic oral vestibular approach	94	SURGERY	4.551	Wang, CC	2014
7	Transoral thyroidectomy and parathyroidectomy—A North American series of robotic and endoscopic transoral approaches to the central neck	87	ORAL ONCOLOGY	5.895	Russell, JO	2017
8	Transoral robotic thyroidectomy: lessons learned from an initial consecutive series of 24 patients	80	SURGICAL ENDOSCOPY AND OTHER INTERVENTIONAL TECHNIQUES	3.796	Kim, HY	2018
9	Transoral endoscopic thyroidectomy vestibular approach (TOETVA) for Graves’ disease: a comparison of surgical	63	GLAND SURGERY	2.55	Jitpratoom, P	2016
10	Learning Curve for Transoral Endoscopic Thyroid Lobectomy	61	OTOLARYNGOLOGY-HEAD AND NECK SURGERY	4.425	Razavi, CR	2018

### 3.4. Keyword and topic clustering analysis to predict future trends

**Figure 4:**
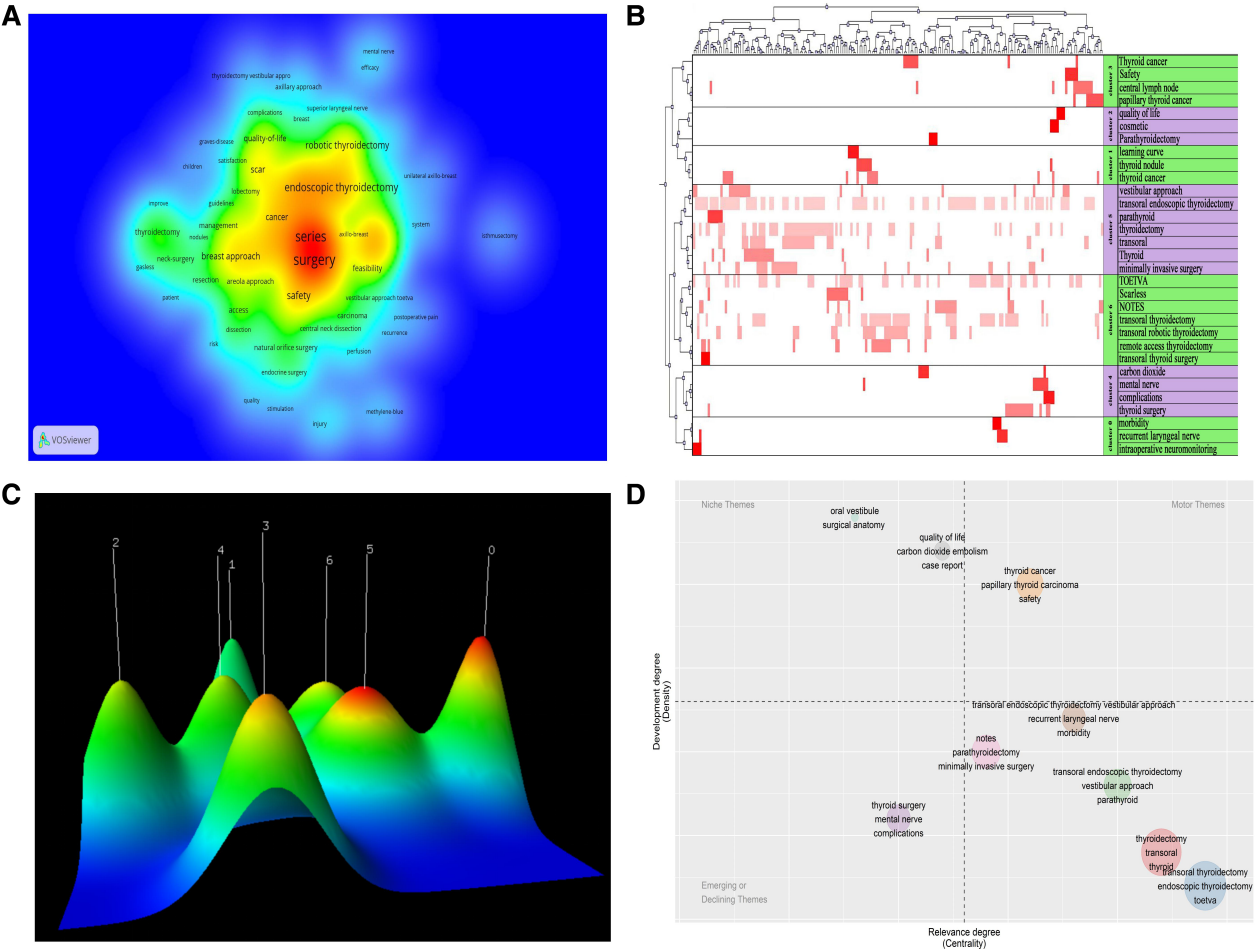
(**A**) the density map of keywords; (**B**) the visualized heat map linked to data matrix of keywords; (**C**) the visualized mountain map of theme clustering; (**D**) the thematic map of keywords (bubble chart).

The high frequency keywords reveal the research focus of scholars in a field. The top 20 high-frequency words from Figure 4A (density plot of keywords) are elucidated as follows: surgery, series, vestibular approach, endoscopic thyroidectomy, outcomes, experience, safety, robotic thyroidectomy, scar, breast approach, video-assisted thyroidectomy, cancer, quality-of-life, approach TOETVA assisted thyroidectomy, initial-experience, parathyroid surgery parathyroid surgery, as well as parathyroidectomy. Figure 4B shows a heat map of the matrix visualization after keyword clustering divided into seven broad clusters: intraoperative monitoring of the laryngeal return nerve (# 0), learning curve (# 1), postoperative quality of life (# 2), central lymph node dissection and safety (# 3), complications (# 4), transoral vestibular pathway is minimally invasive surgery (# 5), and robotic surgery (# 6). Figure 4C shows the visual mountain maps of the seven clusters. After trying different combinations of clusters, it was determined that the 7 clusters had the optimal clustering of the mountain maps, with the peaks being independent of each other and the peaks being brightly colored. Figure 4D provides a graph of TOETVA's theme bubbles. The lower right quadrant is the base theme with a good degree of low centrality development, and themes in this quadrant are currently more maturely researched. The largest number of bubbles in the lower right quadrant reveals that the technology is mature. As depicted in Figure 4A, the themes in the upper right quadrant are the motor themes, i.e., the safety of TOET. This theme is highly central and has a good degree of development, and more scholars will continuously optimize intraoperative and postoperative safety in the future.

## 4. Discussion

The field of TOET has developed significantly over the last decade, especially after 2017 when it entered a rapid development phase and became a popular technology. It has been widely favored by doctors and patients. Accordingly, the hotspots and trends of TOET should be summarized through bibliometrics. This study can provide head and neck surgeons with research highlights and hotspots in the field of TOET. Keeping abreast of developments in relevant studies will be conducive to identifying hotspots for this research and guiding further developments in this field.

Sublingual transoral access for thyroid surgery was first proposed by Entezami P et al. (8). The first application of complete trans-oral video-assisted thyroidectomy (TOVT) to humans was reported by Benhidjeb T in Germany (9). Thomas from Germany reported cases where the use of sublingual transoral access hindered surgical manipulation due to narrow access (10) and increased risk of infection.

Afterward, Wang et al. first reported TOETVA in human patients in 2014 (11). The oral vestibular approach is capable of avoiding damage to the floor of the oral cavity. Moreover, it reduces postoperative damage to the floor of the oral cavity, and it can improve the safety of the procedure. This approach became widespread after Anuwong's 2016 study of 60 patients who underwent the transoral endoscopic thyroidectomy vestibular approach (12), which rank first in the top 10 most cited studies. Subsequently, Anuwong A summarized the postoperative results and surgical indications of TOETVA (13), assessed the safety (6), and significantly optimized the technique. The work of this scholar has been the most cited. Among the top 10 cited studies, except for three highly cited studies by Anuwong, the remaining seven presented TOETVA experience sharing (9, 14), learning curve (15), robotic surgery (16, 17), and inflation-free methods (18). The above 10 highly cited studies have contributed to the development of TOETVA.

In the field of TEOT, there have been frequent exchanges and cooperation between countries. Korea, China and the United States have made outstanding contributions, as manifested by the number of publications and the number of important authors, institutions and journals from the above three countries. Table 3 lists the top ten projects funded by the fund, and they all come from the above three countries. Accordingly, the development of TEOT requires the support of national government funds. Hot topics and future trends in the TOET field can be concluded (e.g., learning curves, surgical points, and complication prevention and management) through clustering analysis of keywords and topics. Considerable studies have indicated the advantages of TOETVA and how to prevent against complications.

**Table 3:** Top 10 funding agencies.

Funding agencies	Funded publications	% of total publications
National Institutes Of Health Nih Usa	7	3.057%
National Research Foundation Of Korea	7	3.057%
Nih National Institute On Deafness Other Communication Disorders Nidcd	7	3.057%
United States Department Of Health Human Services	7	3.057%
National Natural Science Foundation Of China Nsfc	5	2.183%
China Postdoctoral Science Foundation	4	1.747%
Department Of Finance Of Jilin Province	4	1.747%
Department Of Science And Technology Of Jilin Province	4	1.747%
Zhejiang Provincial Basic Public Welfare Project	3	1.310%
Basic Science Research Program Through The National Research Foundation Of Korea Nrf Ministry Of Education Science And Technology	2	0.873%

### 4.1. Intraoperative neuromonitoring of transoral endoscopic thyroidectomy

Intraoperative neuromonitoring (IONM) is considered an important accessory to protect the nerves during conventional radical thyroidectomy (19). Given the high sensitivity and specificity, IONM should be considered a useful tool for thyroid surgery and its use should be suggested for patients undergoing planned total thyroidectomy. Its right application may cancel the risk of bilateral paralysis (20). Memeh K et al. used a doubly robust (DR) estimator in the form of an inverse probability weighted regression adjustment model to estimate the effect of using IONM on the risk of RLN injury and found that the use of IONM was associated with a reduction in RLN injury (21). In a retrospective study by Fei Y et al. divided into IONM and non-IONM groups, the time required to identify the recurrent laryngeal nerve (RLN) was shorter in the IONM group (3.05 ± 1.58 vs. 9.36 ± 4.82 min, *p* < 0.01); the RLN identification rate was much higher in the IONM group than in the non-IONM group (100.00% vs. 88.52%, *p* = 0.01) (22). In our experience, IONM is not only necessary in finding RLN but, at the same time, can help to change some operating habits, such as keeping energy devices away from RLN and using scissors to remove lymph nodes if necessary.

### 4.2. Learning curve

Regarding the learning curve of TORT, many scholars have made studies in this area. Many studies show that intraoperative neuromonitoring help decreased learning curve (23–25), because nerve monitoring can assist in the exploration of the recurrent laryngeal nerve. The learning curve revealed two phases, an initial and a mature phase, for initial phase its range from 15 to 35 cases (26). Chai YJ et al. used cumulative summation (CUSUM) analysis of TOETVA learning curves for glandular lobectomy in 58 cases (27). The learning curves for trans-oral robotic thyroidectomy (TORT) was 25 cases, having a shorter learning curve (28). Meanwhile, *in vitro* simulation training and animal experiments would notably decrease learning curve. Case series from the initial TOETVA operations of four surgeons at three different hospitals were tested over the past few years. Binary logistic regression showed a negative correlation of complication rate and case number (*p* < 0.001, Odds Ratio: 0.91) (29).

### 4.3. Central lymph nodes dissection

Numerous studies have confirmed that TOET can achieve the same effect with open thyroidectomy (OT) in VI and VII neck nodes dissection (5, 30, 31). A recent study indicated that TOETVA outperformed endoscopic thyroidectomy *via* areola approach (ETA) on total central lymph nodes (7.82 ± 3.35 vs. 5.26 ± 2.45, *p* < 0.05) (32). Under the TOETVA viewer, innominate artery can be easily exposed for VII dissection compares to ETA. Zheng, GB et al. also noted that the TOETVA group achieved a higher number of central lymph nodes than the endoscopic thyroidectomy transaxillary approach (ETTA) group (7.2 ± 4.6 vs. 3.9 ± 3.0, *p* < 0.001) (33).

Existing research has initially reported that there was no difference in the number of lymph nodes between the transoral and non-transoral routes, i.e., a result that may have been due to the limited number of studies covered in the early meta-analysis (34). In the latest meta-analysis conducted by Dabsha A et al., trans-oral endoscopic trans-vestibular thyroidectomy (TOT) had an advantage in terms of the number of lymph nodes harvested compared with the trans-axillary route (35). The possible reason for different results of the two meta-analyses is that fewer studies were covered earlier. TOETVA has some advantages in central regional lymph node dissection, because which is a natural route.

### 4.4. Safety and quality of life

Numerous studies have suggested that TOETVA has high safety (36), the amplified visual field has easy to identify recurrent laryngeal nerve and blood vessel, thus improving the safety of the procedure (7, 37). Trans-oral endoscopic treatment can achieve better cosmetic results and a higher quality of life, as compared with the trans-areolar path (38). In our center, no drainage was placed in unilateral thyroidectomy combined with central lymph nodes dissection in common. All those patients recover quickly, and the neck was scarless without swelling. Furthermore, the quality of life related to neck appearance after transoral robotic thyroidectomy is higher than that of conventional open thyroidectomy was verified using 2 questionnaires as follows: the University of Washington QOL questionnaire and the thyroid cancer-specific Quality of Life questionnaire (Thyroid Version) (39). Transoral robotic thyroidectomy is likely to be used by more physicians in the future.

### 4.5. Complication management

Injury of mental nerve was a relative common complication in TOETVA, which can be avoided by preoperative elevation (40, 41). Our experience is that to place the operating TROCA between 3 and 4 teeth firstly and then to place the observe TROCA to prevent mental nerve shift and had accidental injury.

Carbon dioxide (CO2) embolism refers to a rare but potentially devastating complication of in endoscopy surgery. CO2 embolism, characterized by a decrease in end-tidal CO2 and oxygen saturation, can cause rapid intraoperative hypotension and cardiovascular collapse (42). Some reports regarding CO2 embolism in TOETVA, as gas pressure 6 mmhg, this complication would notably decrease. When a CO2 embolism is encountered, my recommendation is to quickly shut off the CO2 and place the patient in a foot-high, head-low position, which will help stabilize the patient's vital signs.

In the early exploration of TOVETA, there were concerns of postoperative infection. As the technology matures, strict oral sterilization or antisepsis can prevent infection after surgery. Previous studies have shown that although TOETVA is technically mature, its learning curve is significantly longer than that of ETA and open surgery, and some complications, such as carbon dioxide embolism, mental nerve injury, infection, skin burn, recurrent laryngeal nerve injury, etc., can be well prevented by skilled surgeons during the operation. Regression analysis of TOETVA complications by Fernandez-Ranvier G et al. showed the most significant reduction in complications in case 12, with the risk and severity of complications decreasing significantly as the number of cases increased (23). In addition, delayed tracheal rupture was reported after 1 week of TOETVA treatment (43). Previous studies have shown judicious use of energy instruments and a safe distance from the windpipe can avoid tracheal damage..

### 4.6. Recent progress

The safety of the procedure was the motor theme from the topic map analysis (Figure 4B), and it is believed that this topic will continue to be a hot topic of research in the future. Transoral robotic thyroidectomy has become popular with a wide range of surgeons over the last three years, and its safety, quality of life and effectiveness have aroused much scholarly attention (28, 44).

A transoral endoscopic thyroidectomy transmandibular approach has recently been reported to be effective and safe, avoiding skin numbness in the central mandibular region and reducing the difficulty of establishing a surgical space (45). In addition, some studies have reported good results with inflation-free TOETVA, such as clear visual fields and no CO2 gas embolism complications (18, 33, 46, 47). As the technology develops, several studies suggest TOETVA may be a feasible and safe thyroidectomy for children (48). The above new findings and perspectives make TOETVA very promising for the future.

### 4.7. Limitations

First, studies from WoS-indexed journals were only analyzed; thus, some novel studies regarding TOET may have been overlooked and excluded from this study. Second, since the classification of studies is based on the number of citations reported, some of the best recently published studies in the field failed to attract our attention due to the low number of citations; therefore, regular bibliometric updates of the study are required. Third, only original published studies were analyzed in this study, and other forms of research (e.g., proposals, conferences, and materials) may not be covered, and new insights may be missed. Although there may be shortcomings, they do not affect TOETVA's comprehensive and exhaustive analysis of the literature in our study.

## 5. Conclusion

This study is dedicated to exploring hot topics and developing features in the field of TOET through a visual bibliometric analysis. TOET's topics primarily focus on learning curves, recurrent laryngeal nerve monitoring, carbon dioxide gas embolism, mental nerve injury, surgical complications, and surgical safety. In the future, more academics will place a focus on the safety of the procedure and reduce the number of complications. Furthermore, more updates may be reported on TOETVA technology and optimization of the safety of the procedure.

## Data Availability

The original contributions presented in the study are included in the article/Supplementary Material, further inquiries can be directed to the corresponding authors.
